# Phylogeography of a Land Snail Suggests Trans-Mediterranean Neolithic Transport

**DOI:** 10.1371/journal.pone.0020734

**Published:** 2011-06-22

**Authors:** Ruth Jesse, Errol Véla, Markus Pfenninger

**Affiliations:** 1 Institute of Ecology, Evolution and Diversity, Goethe University, Frankfurt, Germany; 2 University of Montpellier-2, UMR AMAP, TA A51/PS2, Montpellier, France; 3 Molecular Ecology Group, Biodiversity & Climate Research Centre, Frankfurt, Germany; Natural History Museum of Denmark, Denmark

## Abstract

**Background:**

Fragmented distribution ranges of species with little active dispersal capacity raise the question about their place of origin and the processes and timing of either range fragmentation or dispersal. The peculiar distribution of the land snail *Tudorella sulcata s. str.* in Southern France, Sardinia and Algeria is such a challenging case.

**Methodology:**

Statistical phylogeographic analyses with mitochondrial COI and nuclear hsp70 haplotypes were used to answer the questions of the species' origin, sequence and timing of dispersal. The origin of the species was on Sardinia. Starting from there, a first expansion to Algeria and then to France took place. Abiotic and zoochorous dispersal could be excluded by considering the species' life style, leaving only anthropogenic translocation as parsimonious explanation. The geographic expansion could be dated to approximately 8,000 years before present with a 95% confidence interval of 10,000 to 3,000 years before present.

**Conclusions:**

This period coincides with the Neolithic expansion in the Western Mediterranean, suggesting a role of these settlers as vectors. Our findings thus propose that non-domesticated animals and plants may give hints on the direction and timing of early human expansion routes.

## Introduction

Ever since people have had the ability to move around the planet, they have been introducing new species, either intentionally or accidentally, into new geographical areas [1]. Likewise, the biodiversity in the Mediterranean Basin was shaped, at least since the Neolithic emergence (ca. 12.000–3.000 years before present) by human landscape management and species introductions which usually impacted the indigenous fauna and flora [2]. Since the beginning of live-stock domestication about 10.000–9.500 years before present (BP) in the Eastern Mediterranean, approximately a millennium after the first domestication of crop plants, both were spread westwards with settlers disseminating the Neolithic culture. From then on, the Mediterranean region has served as sink and source for extensive exchange of biodiversity associated to human use [2].

But also species not directly involved in human exploitation were spread accidentally from the very beginning of human migrations, like weed propagules contaminating crop seeds or blind passengers in ship ballasts [1]. In the Mediterranean, it is known that at least nine land snail species were dispersed as an unintentional by-product of bronze-age maritime copper and resin trade [3]. Analysis of land snail shells found in a ship wreck indicated that they were carried off alive with scrub used to cushion heavy freight [3].

The possibility of (un)-intentional introductions in addition to natural range expansions of a species makes it often difficult to infer the direction and timing of dispersal events [4]. Historic records of first sightings of a species in a new area are rare [5], in particular for organisms that are inconspicuous, not a pest or otherwise attracting human attention. Moreover such records are, by definition, non-existing for prehistoric times. In these cases, the (sub)fossil archaeological record can provide an estimate of the minimum time of residence in an area [6], [7]. However, not all organisms are prone to fossilisation or their remains cannot be attributed to a particular species with the necessary certainty. To overcome these difficulties, various methodological approaches based on the distribution of genetic variation have been developed in the past 25 years. The rationale behind these phylogeographic approaches is the reconstruction of the demographic history of the extant populations from DNA sequence data [8].

The distribution of the land snail *Tudorella sulcata s. str.* in Southern France, Sardinia and Algeria - areas that are currently separated by an ocean and that were last in direct contact about 30 million years ago - raises the strong suspicion that this species could have been at some point in the past subject to passive dispersal. The rather large snail (15–20 mm height) has a low active dispersal capacity and a covert life style, burrowed mostly in the soil under rocks and shrubs, except for activity phases during wet weather periods [9]. The first record of the species in literature is its description from Provence by Draparnaud in 1805, thus setting the minimum age for the introduction to Southern France. Due to the presence of cryptic species, it is unfortunately not possible to attribute fossilised shells to extant species in the genus *Tudorella* [9], [10]. Consequently, we used statistical phylogeographic analyses to successfully answer two major questions:

- Where is the origin of the land snail species *Tudorella sulcata* and what was the colonisation sequence?
- When were the respective areas invaded?

## Methods

### Molecular analyses

**Figure 1:**
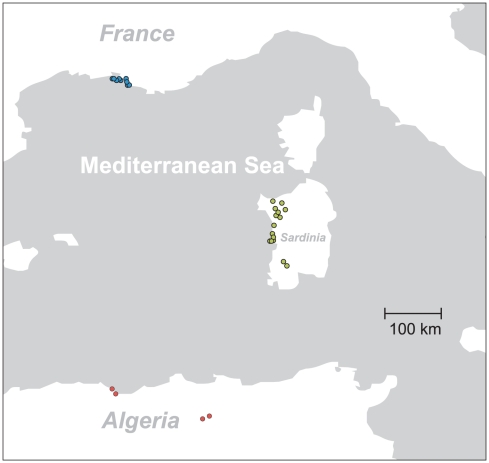
Sampling site distribution. The sampling sites comprise all known occurrences of *T. sulcatus* s.str.. Sampling sites in France are blue, Algeria red and Sardinia green, indicating pooling for gene-flow analysis.

The present study covered all known occurrences of the species *Tudorella sulcata sensu stricto* (Draparnaud 1805) as described in Pfenninger *et al.* [10] (Figure 1, Table S1). For each of the 28 sampled locations, at least 5 apparently living individuals were preserved in 90% alcohol. Further information about the sampling sites is given in the additional files.

For DNA extraction a part of the foot muscle was taken. Genomic DNA was extracted using the CTAB method. For 138 individuals, a 604 bp segment of the cytochrome oxidase subunit I gene (COI) was PCR-amplified. PCR conditions and primers were adopted from the study of Pfenninger *et al.* [10].

Additionally, a 219 bp fraction of the nuclear coded heat shock 70 kD protein (hsp70) was sequenced. The primers with the sequences HSP71Iifor TGCAGCATCTTCTGGATACG and HSP71Iirev GCTCTACGGCGTCTACGAAC were used for PCR amplification under the same conditions as for COI.

PCR products were purified using Pure Link™ PCR Purification Kit (invitrogen, Carlsbad, CA, USA). Ten nanograms per sample were subjected to forward and reverse cycle sequencing using the ABI Prism Big Dye terminator kit (Perkin-Elmer, Norwalk, CT, USA). Sequencing reactions were electrophoresed on an ABI 377 automated DNA sequencer.

As the nuclear hsp70 locus is diploid, we checked the trace files for dinucleotide ambiguities. We counted a site as heterozygous single nucleotide polymorphism when the same ambiguity occurred in both forward and reverse sequencing trace file. We inferred the haplotype phases of heterozygous individuals with the coalescent-based Bayesian method PHASE 2.1 [11] as implemented in DNAsp 5.0 [12].

### Haplotype phylogeny

Sequences were aligned with Clustal W [13] and manually adjusted. Statistical parsimony (SP) cladograms were constructed for both genes separately using TCS vers. 1.21 [14] with the connection limit was set at 95%.

### Phylogeographic Model Selection (PMS)

To compare the relevance of nine explicit dispersal hypotheses, we applied a model selection approach [15], [16], introduced into phylogeography by Pfenninger & Posada [17] and further developed in Depraz *et al.* [18]. The 28 and 17 sampling sites for mitochondrial and nuclear marker, respectively, were pooled into three geographic groups (France (F), Sardinia (S) and Algeria (A), Figure 1). Taking each of the three regions as potential origin in turn, we evaluated i) the hypothesis that each of the other two regions were independently colonised from there and ii) that either one of the two regions was colonised first and the remaining region from there. This resulted in nine hypotheses, which were translated into the corresponding gene-flow matrices. The analyses were carried out for the mitochondrial and nuclear fragment separately.

The maximum likelihood migration rate matrix of each model was then estimated using migrate-n version 2.3 [19]. The first genealogy was started with a random tree. Initial theta and migrant values were generated from an F_ST_ calculation. A static heating scheme with four different temperatures was applied. We ran ten short chains with 4×10^4^ generations each, from which 1000 trees were recorded in regular intervals after a burn-in phase of 20.000 generations. These were followed by three long chains of 10^5^ generations, from which 10^3^ trees were sampled after a burn-in period of 2×10^3^ generations. Parameter estimates were gained from the last chain. Log likelihood estimates cannot be directly compared over different runs with migrate-n. We therefore ran a final analysis with an unconstrained migration model using the likelihood-ratio-test option to gain likelihood estimates that were comparable between the different hypotheses and their parameter sets. We used these estimates and the number of free parameters in each model to calculate the Akaike Information Criterion (AIC, [20]).

### Demographic analysis

Dating demographic expansions associated with geographic expansions was performed with the extended version of the Bayesian Skyline Analysis [21] implemented in BEAST 1.5.2 [22] for the COI fragment only, because no molecular clock estimate was available for the hsp70 locus. A skyline plot is a model of population size fitting a wide range of demographic histories. The Bayesian skyline model uses standard Markov chain Monte Carlo (MCMC) sampling procedures to estimate a posterior distribution of effective population size through time from gene sequences, given a model of sequence evolution. If a molecular clock rate is known for the sequence in question, the model can be used to put demographic events into a historical context [21]. As we are dealing with intraspecific data, where systematic rate heterogeneity is not expected, we have chosen a strict molecular clock model with a fixed rate of 4.28×10^−8^ changes per site and year, as estimated specifically for this *Tudorella* lineage [10]. Even though a strictly intraspecific calibration point would be more desirable, the use of a rate estimated for a terminal branch avoids the overestimation of divergence dates due to saturation associated with calibration points from deep interspecific nodes [23].

A series of initial runs performed to optimise priors and run-time parameter choice indicated that it was necessary to run 4×10^7^ generations of the Monte-Carlo-Markov-Chain, sampling every 1000th generation, to obtain effective sampling sizes above 500 for all estimated parameters. We have chosen the General Time Reversible model with empirical base composition as site evolution model, a gamma distribution of rate heterogeneity with four rate categories and invariant sites, because initial runs indicated that no alternative models fitted the data significantly better. As tree model, the Extended Bayesian Skyline Model for mitochondrial data with linear growth between population size change events was applied with a UPGMA generated tree as starting point. The prior for the number of population size changes was a Poisson distribution with a mean of two, as we were expecting two expansion events (see results). A uniform distribution between 10^4^ and 10^12^ with an initial size of 10^6^ was set for the demographic population mean prior, as no information on the actual population size of *T. sulcata* was available. We tested the skyline model against a model of constant population size, using otherwise the same parameters and priors. The models were compared using log Bayes factors.

## Results

### Phylogenetic relationships

**Figure 2:**
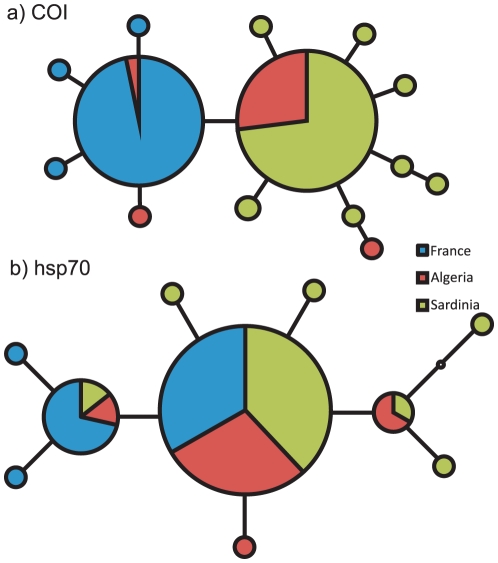
Statistical parsimony networks with based on 604 bp of the mitochondrial COI gene (a) and 219 bp of the nuclear hsp70 gene (b). Circles depict sampled haplotypes, their size is proportional to their frequency. Connecting lines correspond to single mutational steps. Haplotypes occurring in France are blue, Algeria red and Sardinia green.

We obtained 138 COI sequences of 604 bp length (Table S1). All sequences aligned unambiguously and translated without stop codons. Statistical parsimony analysis resulted in a network of 13 haplotypes, defined by 12 singleton mutations and five parsimony informative (PI) sites (Figure 2a). Haplotype diversities for the three geographical regions are: Sardinia 0.160, Algeria 0.143 and France 0.067. Haplotype sequences are registered in GenBank, accession numbers are GU385953–GU385966.

For the hsp70 locus 38 individuals were typed (Table S1). The majority of sequences contained one or more ambiguous sites which indicated the presence of heterozygous individuals. We inferred ten different haplotypes (GenBank acc. nos. JF967635–JF967644), defined by two PI and 8 singleton sites (Figure 2b). The region with the highest haplotype diversity for this marker was Sardinia (0.005), followed by Algeria (0.004) and France (0.003).

### Inference of dispersal routes

Model selection criteria preferred the S>A>F model for both loci (AIC = −33.2, LnL = 21.6 and AIC = −379,78, LnL = 194,89, respectively) over the remaining eight models (Table 1). The best supported model implies that the origin of the expansion was on Sardinia. Starting from there a first expansion to Algeria and a second expansion from Algeria to France took place.

**Table 1:** Phylogeographic model testing.

		COI		hsp70	
Scenario	No. of parameters	LnL	AIC	LnL	AIC
full model	9	21.6	−25.2	194.88	−371.76
S>A, S>F	5	−6.0	22.0	−44.58	99.15
F>S, F>A	5	−63.9	137.9	−318.35	646.70
A>F, A>S	5	−37.7	85.4	−74.56	159.12
F>S>A	5	−6.0	22.0	−74.56	159.12
A>S>F	5	−64.0	138.0	−314.03	638.06
S>F>A	5	−63.9	137.9	−290.15	590.30
A>F>S	5	−37.7	85.4	−71.33	152.66
S>A>F	5	21.6	−33.2	194.89	−379.79
F>A>S	5	−55.6	121.2	−318.35	646.70

Results of the migration hypothesis model selection for mitochondrial COI and nuclear hsp70 locus between the regions Sardinia (S), Algeria (A) and France (F). AIC values measure the fit of the models to the data, taking different parameterisation into account. Note that smaller values indicate better fit. Models suggesting origin in Sardinia, followed by expansion to Algeria and subsequent colonisation of France (S>A>F) received decisive support (ΔAIC>55.2 and 478.94, respectively).

### Demographic analyses

The median estimate in the Bayesian skyline plot analysis for the beginning of population growth was approximately 8.000 years BP with a 95% confidence interval ranging from 10.000 to 3.000 years BP. Figure 2 shows the estimated increase of effective population size with 95% confidence intervals. The skyline model inferring a population expansion was decisively better than a constant population size model (log Bayes factor = 3.64).

## Discussion

Statistical phylogeography is a powerful tool to answer questions about a species' origin, dispersal routes and timing of expansions in a rigorous statistical framework and has been successfully established in the last years [4], [24], [25]. However, such analyses demand a sampling of the whole range of the focal species. The distribution range of *Tudorella sulcata* comprising Southern France, Western Sardinia and Algeria, is well know due to a previous study [10] on the range wide analysis of the genus and could thus be comprehensively sampled.

### Expansion sequence

The occurrence of Algerian mitochondrial haplotypes both on Sardinia and in France and the lack of shared haplotypes between France and Sardinia argue either for an Algerian origin or a connection of the areas through this country (Figure 2a). The hsp70 haplotypes showed a very similar distribution pattern. However, as expected for a nuclear locus with deeper coalescent times, most haplotypes are shared between regions (Figure 2b).

To distinguish among all nine possible expansion sequence models by measuring the fit of the data to the models, we relied on phylogeographic model selection. The advantage of this alternative to classical hypothesis testing is the possibility to evaluate several models simultaneously instead of testing repeatedly single hypotheses against a null model [15]. Analyses of both independently inherited marker strongly supported an origin of *Tudorella sulcata* on Sardinia and an expansion from there first to Algeria and subsequently to France (Table 1). It is thus likely that our results reflect the true population history and are not due to the particular coalescence history of the respective genes.

Sardinia as origin of the species was also indicated by the highest haplotype diversity for both mitochondrial and nuclear marker in that region. The lack of support for France as origin of the species is also compatible with the results of a biogeographical analysis that excluded France as possible origin for this species [10].

The somehow counter-intuitive dispersal sequence suggested by our analysis, in addition to the species' biology makes a natural expansion very unlikely. Dispersal by log-rafting in the sequence Sardinia-Algeria-France is most implausible when considering the surface circulations in the Western Mediterranean Sea. Dense water from the Tyrrhenian Sea is transported in a current flowing from Sardinia northwards to Corsica and France and counter clockwise along the French and Spanish coasts until reaching Northern Africa near the Strait of Gibraltar [26]. The Algerian Current is going 3–5 km per day eastwards in anticlockwise circulation along the northern continental slope, making drift from Algeria to France under regular conditions highly unlikely as it would require the snails to survive a several week long journey exposed to saltwater.

The maximum reported dispersal distance of light objects (<450 g) by extreme strong winds is 130 km [27]. As the distances between Sardinia and Algeria (ca. 250 km) as well as Algeria and France (ca. 700 km) exceed this maximum by far, it is reasonable to dismiss the hypothesis of wind-borne dispersal.

The pattern observed in our analysis thus suggests transportation through biological vectors. Migrating birds are potential over-sea vectors for small invertebrates, but rather for freshwater species [28], [29]. Usually eggs or larval stages are transported, attached to feathers of water fowl. *Tudorella sulcata* lives mostly buried in the soil and contact to migrating birds is hardly imaginable. Even though important bird migration routes run between central Africa and Europe, transport by birds is thus unlikely for this land snail and would not explain the colonisation sequence.

An ancient expansion (e.g. during the Messinian Salinity Crisis 5.96±0.20 Mya [30] was a priori unlikely in the light of the shallow divergence of the populations and the COI molecular clock rate estimated from phylogenetic analysis [10]. After exclusion of the possible alternatives, anthropogenic passive dispersal remains as sole plausible mechanism.

### Timing of the expansion

**Figure 3:**
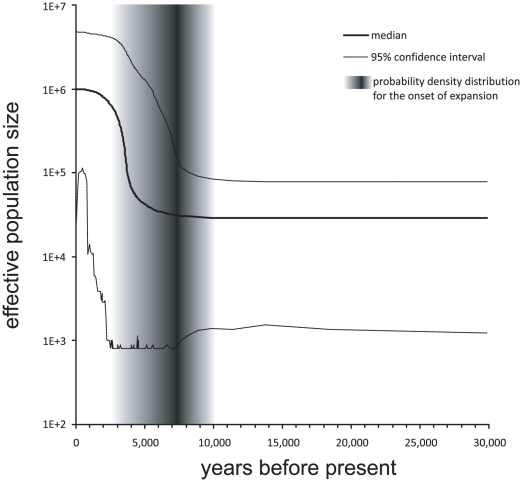
Bayesian skyline plot. Historical development of population sizes through time based on Bayesian coalescence analyses of the COI locus. The analysis suggests a population increase between 3,000 and 10,000 years bp with the highest probability density around 8,000 years bp.

As a colonisation of new areas is logically linked to demographic expansion, the dating of the latter can give a time frame for these events. Bayesian skyline plot estimates suggested that a population expansion in *Tudorella sulcata* started between 10.000 and 3.000 years ago (Figure 3). This broad interval excludes historic times on one end and Pleistocene dispersal on the other. The highest probability density of the estimate lay around 8000 years BP. This period coincides on the one hand with the beginning of the Neolithic period in the Western Mediterranean and on the other with the Holocene climate optimum [31]. The latter may have been causal for the onset of the observed population expansion, just like the postglacial warming was for the spatial and demographic expansion of several land snail species [17], [18], [32]. However, if this climate change was indeed triggering a major population dynamic in *T. sulcata*, the subsequent cooling starting around 4000 ybp should have also left its mark as population decline. Additionally, a climate driven population size increase would not explain the transoceanic dispersal.

The Holocene optimum may have nevertheless played an indirect role for the colonisation of new habitats by triggering or facilitating the Neolithic period [33]. Coming from the Near East (Levant), Neolithic colonists started around 10.500 years BP to migrate first into the Balkans and then continuously colonised the European continent in expansion waves westwards during the following 4.500 years, with a peak of migration around 8.000 years BP [2], [34], [35]. First sea crossings in the Mediterranean are documented already for the Mesolithic Age by obsidian originating from the Cycladic island of Melos found in Mesolithic sediments of the Franchthi cave on the Peloponnesus [36]. Numerous obsidian artefacts found in Italy, Southern France, Eastern Spain, Croatia, Greece, Tunisia and Algeria from far away sources document the efficiency of trans- oceanic transport during the Neolithic period [37]. The synchrony of the demographic expansion with this phase of early human expansion and the exclusion of other factors suggests indeed that Neolithic settlers or traders were acting as vectors for the snails. Genetic analyses of Sardinian human populations connect Sardinia and Northern Africa through early human migrations and strengthen the above scenario [38], [39].

The secondary expansion from Algeria to France is more difficult to date because the installation of the rather small population there has not left discernable traces like a steepening slope in the demographic reconstruction curve even though it contributed to the general population size increase. In Southern France, the first Neolithic coastal settlements date to 7.700–7.600 years BP [2]. Contacts between the French and Algerian coasts have been numerous since then. However, the private haplotypes found in France (Figure 1a+b) argue for a relatively early introduction.

Without further archaeological evidence, it is impossible to determine the purpose the snails were transported for. Unintentional shipping as in the case of the Uluburun wreck [3], transportation as trading good or simply because the light pink, regularly sculptured shells have a quite attractive appearance, can be imagined.

Passive dispersal of *Tudorella* species across marine barriers to the nearest opposite coasts appear to have happened several times [10]. The presence of the same species in Morocco and Southern Spain (*T. mauretanica*) as well in Tunisia and Sicily (*T. multisulcata*) illustrate this [10]. However, in these cases the Pleistocene sea level changes [40] may have facilitated short range human or animal passive transport or may have even allowed active dispersal between these regions.

### Conclusions

This study indicates that the population histories of non-domesticated animals and plants may give hints on the direction and timing of early human expansion routes. It suggests that modern invasions, such as the well-known zebra-mussel proliferation in freshwaters worldwide [41] or the invasion of the Asian tiger mosquito in Europe [42], are only among the latest instances of a long series of anthropogenic introductions, shaping the distribution of extant biodiversity.

## Supporting Information

Table S1 List of sampling sites used in the study.
(DOC)
