# Supplementary material for: Phylogeography of a Land Snail Suggests Trans-Mediterranean Neolithic Transport
Source: PLoS One. 2011 Jun 22;6(6):e20734. doi: 10.1371/journal.pone.0020734 (PMC3120762; doi:10.1371/journal.pone.0020734)
Supplement: Table S1 — List of sampling sites used in the study. (DOC) [file pone.0020734.s001.doc]

## Table S1. Sampling sites.

| Region  Sampling site | Latitude | Longitude | Number of COI haplotypes | Number of hsp70 haplotypes |
| --- | --- | --- | --- | --- |
| France |  |  | 60 | 28 |
| Anse de Bonnieu | N 43° 20,668' | E 5° 01,407' | 5 | 4 |
| Bonnieu | N 43° 20,700' | E 5° 01,533' | 7 | 4 |
| Couroune | N 43° 19,700' | E 5° 04,450' | 7 | 4 |
| La Redonne | N 43° 20,127' | E 5° 11,906' | 6 | - |
| Mejean | N 43° 19,857' | E 5° 13,162' | 2 | - |
| Port Miou | N 43° 12,367' | E 5° 30,758' | 6 | 4 |
| Resquiadou | N 43° 21,250' | E 5° 16,833' | 3 | - |
| Roucas-Blanc | N 43° 16,467' | E 5° 22,550' | 6 | 4 |
| Sausset les Pins | N 43° 19,800' | E 5° 05,790' | 7 | 4 |
| Sugiton | N 43° 12,917' | E 5° 26,750' | 11 | 4 |
| Algeria |  |  | 28 | 20 |
| Bejaia | N 36° 45,279' | E 5° 04,568' | 8 | 8 |
| El Khroub | N 36° 16,283' | E 6° 54,367' | 1 | 4 |
| Yemma Gouraya | N 36° 46,167' | E 5° 05,033' | 19 | 8 |
| Sardinia |  |  | 50 | 28 |
| Capo Mannu | N 39° 01,859' | E 8° 22,765' | 4 | - |
| Florinas | N 40° 39,370' | E 8° 38,610' | 2 | - |
| Genna Maria | N 39° 37,944' | E 8° 51,284' | 5 | 4 |
| Is Arenas | N 40° 03,985' | E 8° 30,025' | 4 | 4 |
| Is Arutas | N 39° 57,194' | E 8° 24,390' | 3 | - |
| Lago del Cuga | N 40° 36,825' | E 8° 30,402' | 3 | 4 |
| Mara | N 40° 27,241' | E 8° 35,580' | 4 | - |
| Mari Ermi | N 39° 57,884' | E 8° 26,333' | 2 | 4 |
| Morgongiori | N 39° 42,209' | E 8° 45,084' | 3 | 4 |
| Padria | N 40° 24,240' | E 8° 38,130' | 3 | - |
| Romana | N 40° 28,645' | E 8° 36,120' | 2 | - |
| San Marco | N 40° 11,830' | E 8° 28,860' | 4 | 4 |
| Santa Catarina | N 40° 06,550' | E 8° 29,310' | 4 | - |
| Thiese | N 40° 31,770' | E 8° 42,160' | 3 | 4 |
| Zuchinu | N 40° 43,760' | E 8° 25,880' | 4 | - |

Sampling points of *T. sulcatus* samples, their geographic position and number of individuals analysed for the mitochondrial COI gene and the nuclear hsp70 locus, respectively.
